# *Ixeris dentata* NAKAI Reduces Clinical Score and HIF-1 Expression in Experimental Colitis in Mice

**DOI:** 10.1155/2013/671281

**Published:** 2013-09-08

**Authors:** Dae-Seung Kim, Jang-Ho Ko, Yong-Deok Jeon, Yo-Han Han, Hyun-Ju Kim, Amrit Poudel, Hyun-Ju Jung, Sae-Kwang Ku, Su-Jin Kim, Sang-Hyun Park, Jin-Han Park, Byung-Min Choi, Sung-Joo Park, Jae-Young Um, Seung-Heon Hong

**Affiliations:** ^1^Department of Oriental Pharmacy, College of Pharmacy, Wonkwang-Oriental Medicines Research Institute, Wonkwang University, Iksan, Jeonbuk 570-749, Republic of Korea; ^2^Department of Pharmacology, College of Korean Medicine, Institute of Korean Medicine, Kyung Hee University, Dongdaemun-gu, Seoul 130-701, Republic of Korea; ^3^Department of Anatomy and Histology, College of Oriental Medicine, Daegu Hanny University, Yugok-Dong, Kyungsan 712-715, Republic of Korea; ^4^Department of Cosmeceutical Science, Daegu Hanny University, Yugok-Dong, Kyungsan 712-715, Republic of Korea; ^5^Isotope Sciences Lab, Korea Atomic Energy Research Institute, 1266 Shinjeong-dong, Jeongeup, Jeonbuk 580-185, Republic of Korea; ^6^Department of Medicinal Herb, Gyeongju University, Gyeongbuk 780-712, Republic of Korea; ^7^Department of Biochemistry, School of Medicine, Wonkwang University, Iksan, Chonbuk 570-749, Republic of Korea; ^8^Department of Herbology, College of Oriental Medicine, Wonkwang University, Iksan 570-749, Republic of Korea

## Abstract

*Ixeris dentata* (ID) is an herbal medicine used in Asian countries to treat indigestion, pneumonia, hepatitis, contusions, and tumors; however, its effect on intestinal inflammation is unknown. Thus, we investigated the effect of ID in the dextran sulfate sodium (DSS) model of colitis in female BALB/c mice; animals were evaluated after seven days of DSS treatment. DSS-treated mice showed considerable clinical signs, including weight loss, reduced colon length, colonic epithelial injury, infiltration of inflammatory cells in the colon tissue, and upregulation of inflammatory mediators. However, administration of ID attenuated body weight loss, colon shortening, and the increase in disease activity index score. ID also significantly decreased the colonic mucosal injury and the number of infiltrating mast cells. Moreover, ID inhibited the expressions of cyclooxygenase-2 and hypoxia-inducible factor-1**α** in colon tissue. Taken together, the results provide experimental evidence that ID might be a useful therapy for patients with ulcerative colitis.

## 1. Introduction

Ulcerative colitis (UC) is a chronic inflammatory disorder of the colon and rectum with intervals of acute exacerbation. Its etiology remains unknown, although results from recent studies suggest that proinflammatory cytokines initiate the inflammatory response [1]. Patients with UC have been reported to have increased levels of interleukin- (IL-) 6 in the intestinal mucosa [2, 3] and of tumor-necrosis-factor- (TNF-) *α* in blood, colonic tissue, and stool [4]. Two cyclooxygenase (COX) isoenzymes have been recognized: COX-1, a constitutive enzyme, which generates prostaglandins (PGs) that protect the stomach and kidney against damage, and COX-2, an inducible enzyme induced by inflammatory stimuli, such as cytokines, and capable of generating PGs that contribute to the pain and swelling of inflammation [5, 6]. The expression of COX-2 is also elevated in the inflamed mucosa of patients with UC [7]. Moreover, metabolism is altered in inflamed mucosal tissues, secondary to decreased mucosal perfusion caused by infiltration of inflammatory cells. The resultant hypoxia [8, 9] activates hypoxia-inducible-factor- (HIF-) 1, a transcription factor that links inflammatory pathways [10, 11].

Although corticosteroids are effective in bringing about clinical remission, severe adverse effects can sometimes lead to their discontinuation; thus, alternative treatments are needed. *Ixeris dentata *NAKAI (ID) is a traditional herbal medicine used in Korea to treat indigestion, pneumonia, hepatitis, and tumors [12]. It has also been reported to protect against kainic-acid-induced oxidative stress in the mouse brain by regulating glutathione concentration [13] and to inhibit the anaphylactic response induced by compound 48/80 or IgE [14]. Moreover, lactic acid fermentation of ID increased its potency against IgE-induced allergic diseases [15]. However, it is unknown whether ID can reduce intestinal inflammation.

The dextran sulfate sodium (DSS) model of UC has been well characterized morphologically and biochemically. DSS induces an acute colitis characterized by bloody stools, ulcerations, and infiltration of inflammatory cells [16]. Histologically, DSS produces submucosal erosions, crypt abscesses, and epithelioglandular hyperplasia. It is generally believed that DSS is directly toxic to gut epithelial cells of the basal crypts and affects the integrity of the mucosal barrier [17]. Hence, the DSS-induced colitis model is particularly useful for studying the contribution of inflammatory mechanisms in colitis. To provide experimental evidence that ID might be a useful therapy for patients with UC, we examined the effects of ID on DSS-induced colitis. The specific aims were to assess the effect of ID on clinical signs of colitis, including weight loss, colon shortening, diarrhea, and occult/gross bleeding, and to investigate the effect of ID on proinflammatory mediators in the colon of DSS-treated mice.

## 2. Materials and Methods

### 2.1. Animals and Reagents

Female BALB/c mice (6 weeks old) were obtained from Da-Mool Science (Taejeon, Republic of Korea). Mice were acclimatized in a specific pathogen-free environment under controlled conditions (22 ± 2°C under a 12 h light/dark cycle) for at least one week. All animal studies were carried out in accordance with the regulations issued by the Institutional Review Board of Wonkwang University (confirmation number: WKU11-10). DSS (mol wt: 36,000–50,000) was purchased from MP Biomedicals (Solon, OH, USA). Purified anti-mouse IL-6, recombinant mouse IL-6, and biotinylated anti-mouse IL-6 antibodies were obtained from BD-Pharmingen (San Diego, CA). Specific antibodies against COX-2, HIF-1*α*, and glyceraldehyde 3-phosphate dehydrogenase (GAPDH) were obtained from Santa Cruz Biotechnology (Santa Cruz, CA). All other chemical reagents were purchased from Sigma (St. Louis, MO).

### 2.2. Preparation of ID

ID extract was prepared by decocting with distilled water for three hours (100 g/L). The residue was filtered, lyophilized, and maintained at room temperature. The yield of dried extract from the starting materials was about 7.8%. Dried extract was diluted in saline and filtered through 0.22 *μ*m syringe filter.

### 2.3. Induction of Colitis by DSS

Acute colitis was induced by administering drinking water containing 5% (w/v) DSS to mice for seven days. Mice were checked daily for the body weight, stool consistency, and the presence of gross bleeding. Animals were randomized to four groups: control (no DSS), DSS, DSS plus ID (100 mg/kg), and DSS plus sulfasalazine (SFZ; 100 mg/kg) as a reference drug. ID and SFZ were diluted with purified water and orally administered once a day during the seven days of DSS treatment, after which time animals were killed.

### 2.4. Disease Activity Index (DAI)

Intestinal disease activity was assessed based on the weight loss, the presence of diarrhea accompanied by blood and mucus, and colonic shortening [18]. DAIs were calculated by scoring weight loss, diarrhea, and rectal bleeding, based on the scoring system (Table 1) described by Murthy et al. [19]. Weight loss was defined as the difference between initial and final weights and diarrhea as the absence of fecal pellet formation and the presence of continuous fluid fecal material in the colon. Rectal bleeding was assessed based on the presence of diarrhea containing visible blood and on the presence of gross rectal bleeding. DAI values were calculated using the following formula: DAI = {(weight loss score) + (diarrhea score) + (rectal bleeding score)}/4. The DAI was determined by three investigators blinded to the protocol. The clinical parameters used in the present study were chosen to represent the subjective clinical symptoms observed in human UC.

### 2.5. Cytokine Assays

Levels of IL-6 and TNF-*α* in the serum and tissue were measured using an enzyme-linked immunosorbent assay (ELISA), as previously described [20]. Briefly, 96-well plates were coated with 100 *μ*L of anti-mouse monoclonal antibodies (1.0 mg/mL at pH 7.4 in phosphate-buffered saline [PBS]) and incubated overnight at 4°C. After additional washes, 50 *μ*L of sample or IL-6 and TNF-*α* standard was added and incubated at room temperature for two hours. Plates were then washed, and 0.2 *μ*g/mL of biotinylated anti-mouse antibody was added and incubated at room temperature for two hours. After washing the plates, avidin-peroxidase was added, and plates were incubated for 30 min at 37°C. The plates were then washed again and ABTS substrate was added. Color development was measured at 405 nm using an automated microplate ELISA reader. Standard curves were prepared using serial dilutions of recombinant antibodies. Protein concentrations were measured using bicinchoninic acid (BCA) protein assay reagent (Sigma). 

### 2.6. Western Blot Analysis

Distal colons were homogenized in lysis buffer (iNtRON Biotech, Republic of Korea) and centrifuged at 13,000 rpm for five min. The supernatants were transferred to fresh tubes, and protein concentrations were determined using BCA protein assay reagent (Sigma). Lysates (50 *μ*g of protein) were separated by 10% SDS-PAGE and transferred to membranes (Amersham Pharmacia Biotech, Piscataway, NJ), which were blocked with 5% skim milk in PBS-Tween-20 (PBST) for 1 h at room temperature. Membranes were incubated overnight with primary antibodies against COX-2 and HIF-1*α* and washed 3 times with PBST. Blots were incubated with secondary antibodies for one hour at room temperature; antibody-specific proteins were visualized using an enhanced chemiluminescence detection system (Amersham Corp. Newark, NJ, USA). Protein densities were quantified by densitometry.

### 2.7. Histological Processing

All trimmed rectums were fixed in 10% neutral buffered formalin. After paraffin embedding, 4 *μ*m sections were prepared. Representative sections were stained with hematoxylin and eosin (H&E) for examination under light microscopy or with toluidine blue to detect mast cells.

### 2.8. Microscopic Scoring

The histological damage on the prepared, cross trimmed H&E stained samples were evaluated by two pathologist observers who were blinded to the experimental groups according to the modified criteria (Table 2) from Hamamoto et al. [21]. Briefly, the mucosa damages were scored 0–4 based on the loss of crypt (mucosa) and infiltration of inflammatory cells (max. score = 4).

### 2.9. Histomorphometry

Thickness of the rectal mucosa (*μ*m/cross trimmed rectum) and numbers of infiltrating inflammatory cells (cells/mm^2^ of mucosa) or mast cells (cells/mm^2^ of mucosa, in toluidine blue stain) in the mucosa were calculated for individual histology samples using a digital image analyzer (DMI-300, DMI, Republic of Korea). 

### 2.10. HPLC Analysis

The chromatographic system consisted of a pump (Gilson, 321 pump) and a UV detector (Gilson, 151 detector). For enhanced separation, a C_8_ (4.6 × 250 mm) column (Watchers, Japan) was used. Acetonitrile : water : acetic acid (15 : 85 : 1.5) was used as the mobile phase in an isocratic manner. Peaks were detected at 254 nm. The injection volume was 10 *μ*L, and flow rate was maintained at 1.0 mL/min. The sample and standard (3,4-dihydroxy cinnamic acid) were dissolved in 50% methanol/water. The sample was prepared with 10 mg/mL of ID. The stock solution at the concentration of 2 mg/mL of 3,4-dihydroxy cinnamic acid was prepared. The solutions were filtered through a 0.2 *μ*m membrane filter.

### 2.11. Statistical Analysis

The results are presented as mean ± S.E.M of at least three independent experiments. Results were analyzed using PASW Statistics 18.0 program. The Student's*t* test was used to determine statistically significant differences. *P* values of <0.05 were considered significant.

## 3. Results

### 3.1. The Effects of ID on Clinical Signs in DSS-Induced Colitis

**Figure 1:**
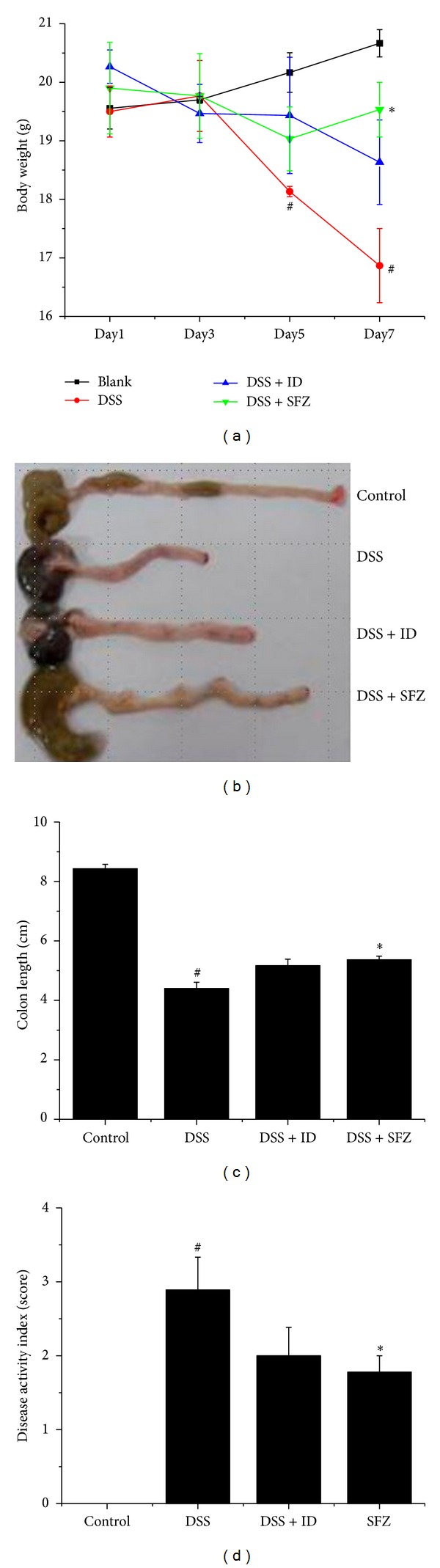
The effect of *Ixeris dentata *NAKAI (ID) on dextran-sodium-sulfate- (DSS-) induced clinical signs. Ulcerative colitis was induced in female BALC/c mice by administering 5% DSS in the drinking water for seven days. Over the same period, ID (100 mg/kg) and the reference compound sulfasalazine (SFZ; 100 mg/kg) were given orally once daily. (a) Body weights were measured at the same time of the experimental days. (b) Colons were harvested on day 7, and colon lengths were measured. (c) Colon lengths in the four study groups. (d) Disease activity index scores in the four study groups. Values represent mean ± S.E.M. (*n* = 5). Data were analyzed by Student's *t* test (^#^ *P* < 0.05 versus control and *P < 0.05 versus DSS alone).

DSS caused a decrease in body weight (Figure 1(a)) and colon length (Figures 1(b) and 1(c)) at day 7 by 19.5% and 47.8%, respectively, compared to the control group. Both ID and SFZ alleviated the DSS effects on body weight loss and colon shortening (Figures 1(a)–1(c)). ID and SFZ also attenuated the DSS-mediated increase in DAI scores (Figure 1(d)).

### 3.2. The Effect of ID on Levels of IL-6 and TNF-*α* in DSS-Induced Colitis

**Figure 2:**
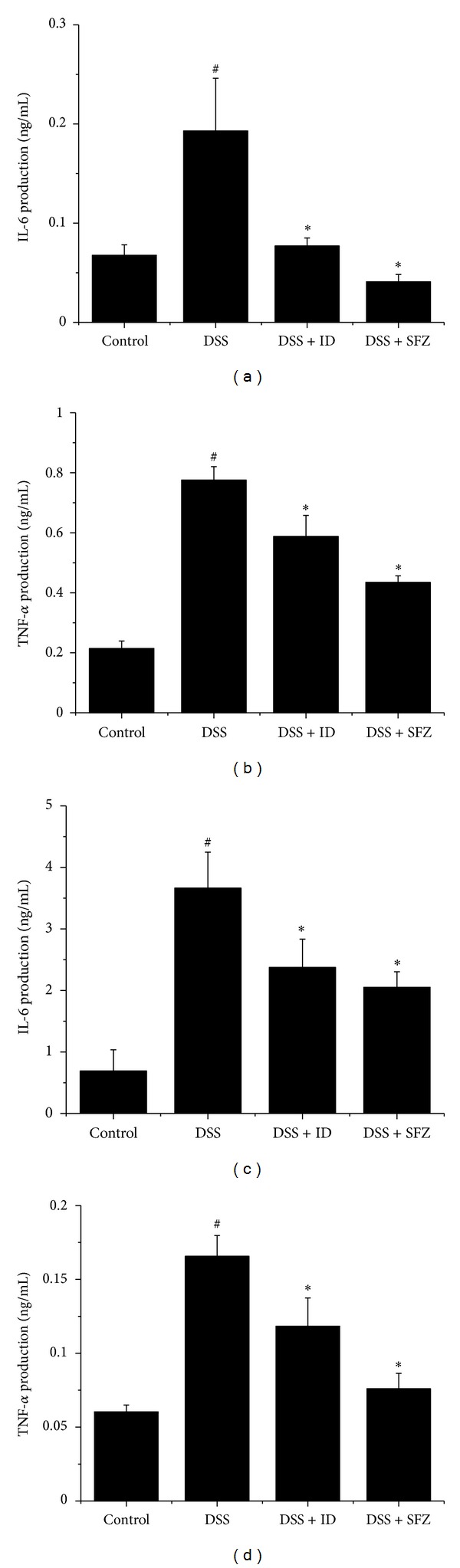
The effect of *Ixeris dentata* NAKAI (ID) on serum levels of interleukin- (IL-) 6 and tumor-necrosis-factor- (TNF-) *α* in DSS-induced colitis. Ulcerative colitis was induced by administering 5% DSS in the drinking water for seven days. Over the same period, ID (100 mg/kg) and the reference compound sulfasalazine (SFZ; 100 mg/kg) were given orally once daily. Cytokine production was determined by ELISA. (a) IL-6 production in mouse serum at day 7. (b) TNF-*α* production in mouse serum at day 7. (c) IL-6 production in colon tissue. (d) TNF-*α* production in colon tissue. Values represent mean ± S.E.M. (*n* = 5). Data were analyzed by Student's *t* test (^#^ *P* < 0.05 versus control and *P < 0.05 versus DSS alone).

Serum IL-6 level was significantly higher in the DSS group (0.193 ± 0.091 ng/mL) than in the control group (0.067 ± 0.018 ng/mL); IL-6 levels were significantly lower in ID (0.077 ± 0.014 ng/mL) or SFZ (0.041 ± 0.013 ng/mL) treatment group (Figure 2(a)). The serum TNF-*α* was also significantly increased in the DSS group (0.776 ± 0.045 ng/mL) compared to control (0.21 ± 0.025 ng/mL); serum TNF-*α* levels were significantly lower in ID (0.558 ± 0.070 ng/mL) or SFZ (0.435 ± 0.022 ng/mL) treatment group (Figure 2(b)). Furthermore, tissue IL-6 and TNF-*α* levels were significantly higher in the DSS groups (3.663 ± 0.585, 1.657 ± 0.140 ng/mL, resp.) than in the control groups (0.690 ± 0.346, 0.603 ± 0.046 ng/mL, resp.); tissue IL-6 and TNF-*α* levels were significantly lower in ID (2.373 ± 0.461, 1.183 ± 0.191 ng/mL, resp.) or SFZ (2.050 ± 0.254, 0.760 ± 0.104 ng/mL, resp.) treatment groups (Figures 2(c) and 2(d)).

### 3.3. The Effect of ID on COX-2 and HIF-1*α* Expression in DSS-Induced Colitis

**Figure 3:**
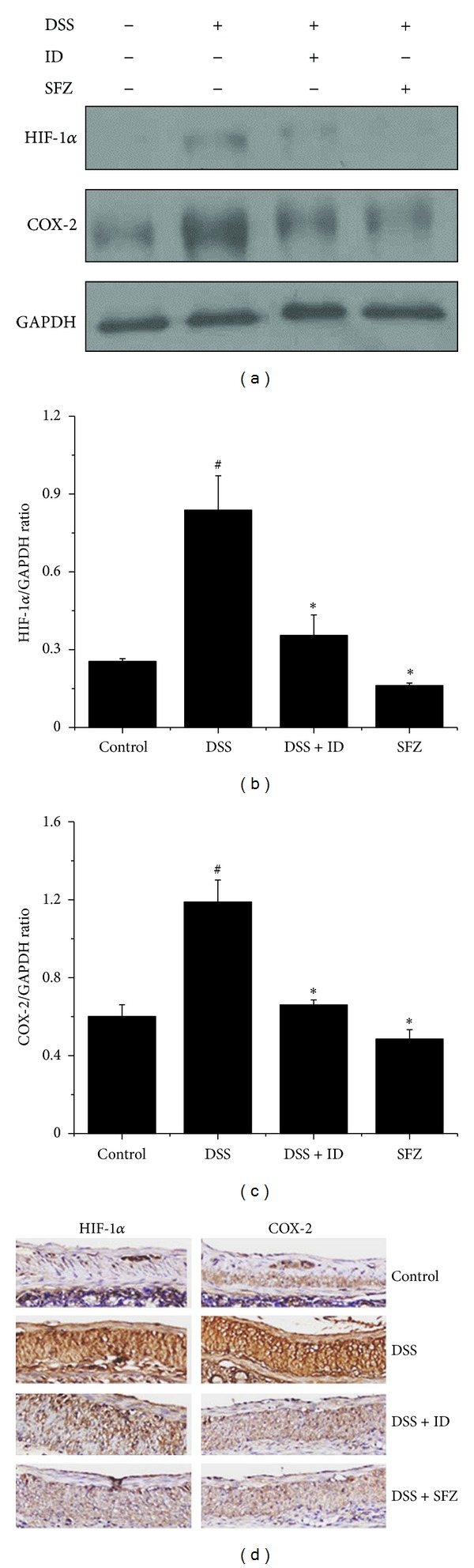
The effect of *Ixeris dentata *NAKAI (ID) on dextran-sodium-sulfate- (DSS-) induced cyclooxygenase- (COX-) 2 and hypoxia-inducible-factor- (HIF-) 1*α* levels in colonic tissues. Ulcerative colitis was induced in female BALC/c mice by administering 5% DSS in the drinking water for seven days. Over the same period, ID (100 mg/kg) and the reference compound sulfasalazine (SFZ; 100 mg/kg) were given orally once daily. COX-2 and HIF-1*α* levels were determined by western blot analysis. (a) Representative western blot (of three independent experiments) of COX-2 and HIF-1*α* expression in colonic tissue. (b) Ratios of COX-2/GAPDH and (c) HIF-1*α*/GAPDH were determined by densitometry. (d) Sections of colons of DSS-treated mice with or without ID treatment were subjected to immunohistochemical analysis. Values represent mean ± S.E.M. Data were analyzed by Student's *t* test (^#^ *P* < 0.05 versus control and *P < 0.05 versus DSS alone).

DSS markedly induced COX-2 and HIF-1*α* expression in colonic tissue versus controls (Figures 3(a)–3(c)); these increases were reduced by ID or SFZ administration. The effect of ID on COX-2 and HIF-1*α* expression was confirmed in colon tissues by immunohistochemical staining (Figure 3(d)).

### 3.4. The Effects of ID on Epithelial Injury and Mast Cells Infiltration in DSS-Induced Colitis

**Figure 4:**
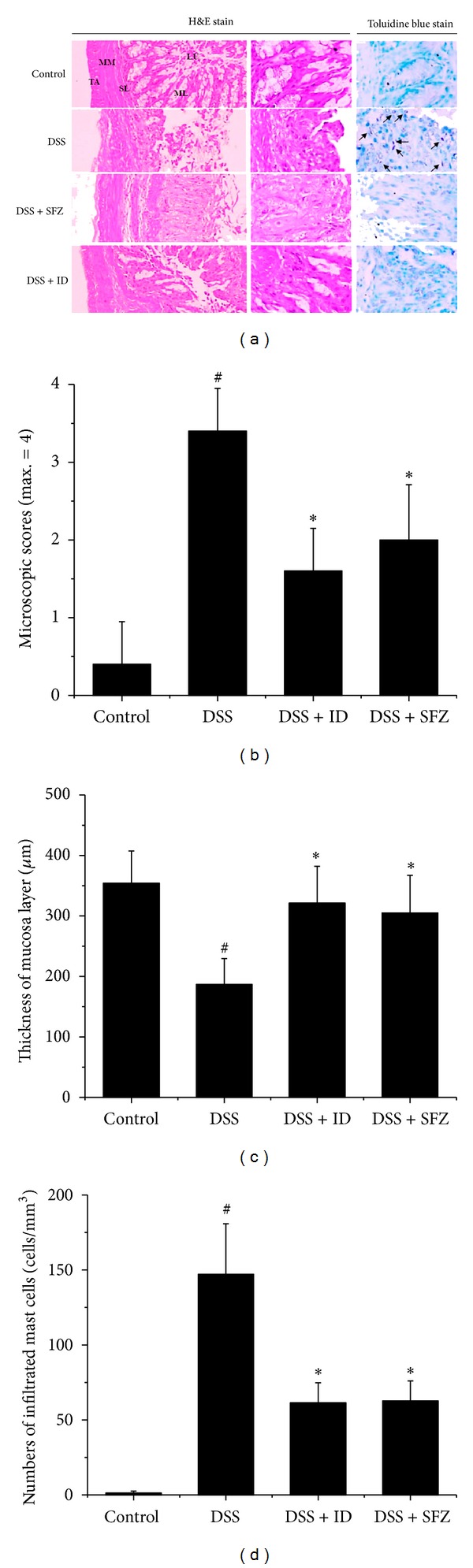
The effects of *Ixeris dentata *NAKAI on epithelial injury and mast cells infiltration in dextran-sodium-sulfate- (DSS-) induced colitis. Ulcerative colitis was induced in female BALC/c mice by administering 5% DSS in the drinking water for seven days. Over the same period, ID (100 mg/kg) and the reference compound sulfasalazine (SFZ; 100 mg/kg) were given orally once daily. (a) Paraffin sections of colonic tissue were stained with hematoxylin and eosin (100x) or with toluidine blue for mast cell identification. Mast cell infiltration is indicated by the arrows (structures: TA, tunica adventitia; MM, muscularis mucosa; SL, submucosa layer; ML, mucosa layer; LU, lumen). Microscopic scores (b), thickness of mucosal layer (c), and number of mast cells (d) were presented. Values represent mean ± S.E.M. (*n* = 5). Data were analyzed by Student's *t* test (^#^ *P* < 0.05 versus control and *P < 0.05 versus DSS alone).

Mucosal thickness is regarded as a parameter of mucosal integrity. DSS treatment caused epithelial injury, as evidenced by an approximate 60% in mucosal thickness (Figures 4(a) and 4(c)). Mucosal infiltration of inflammatory cells, including mast cells (Figure 4(a); arrows), was also detected in the DSS-treated group as compared with the control group (Figures 4(a) and 4(d)). ID and SFZ treatments attenuated these effects induced by DSS treatment (Figures 4(a), 4(c), and 4(d)). ID and SFZ treatments also reduced the DSS-mediated microscopic damage to the colonic tissue (Figure 4(b)).

### 3.5. Characterization of ID Constituents

**Figure 5:**
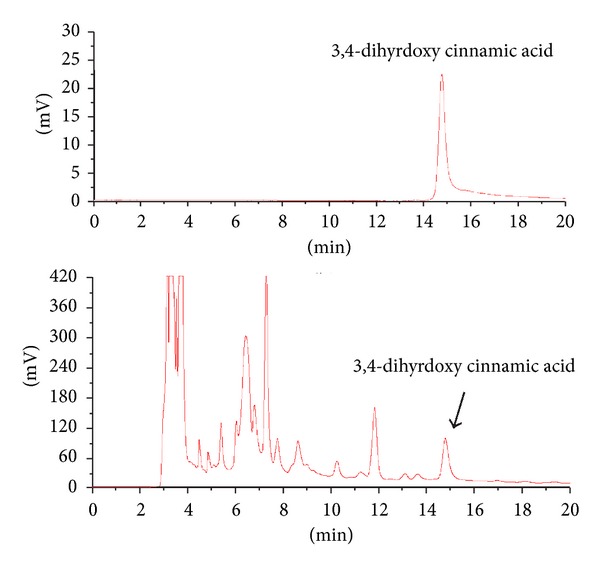
HPLC fingerprints of *Ixeris dentata *NAKAI (ID) and the standard 3,4-dihydroxy cinnamic acid. The mobile phase consisted of acetonitrile : distilled water : glacial acetic acid (15 : 85 : 1.5, isocratic manner). The injection volume was 10 *μ*L of each sample, and flow rate was 1 mL/min. Wavelength was 254 nm. Retention time of 3,4-dihydroxy cinnamic acid was 14.3 min.

A chromatogram of ID along with the standard 3,4-dihydroxy cinnamic acid is shown in Figure 5; the regression (*R* ^2^) of calibration curve was 0.9998. Based on the calibration curve, the content of 3,4-dihydroxy cinnamic acid in ID was estimated to be 4 mg/g of water extract of ID.

## 4. Discussion

UC is a type of inflammatory bowel disease (IBD), the symptoms of which include abdominal pain, weight loss, and bloody diarrhea [22–24]. Most therapies for UC include glucocorticosteroids, sulfasalazine, and immunomodulators (such as azathioprine) [25, 26]; however, these treatments can cause serious adverse effects. Although traditional herbal medicines have garnered much interest for their potential to treat inflammation, their pharmacological mechanisms of action have remained largely unresolved. Here it is demonstrated that ID alleviates the clinical signs—weight loss, colon shortening, diarrhea, and occult/gross bleeding—in the DSS-treated mouse model of UC. We also found that ID reduced epithelial injury, inflammatory cell infiltration into the colon tissue, and indices of microscopic injury. Moreover, ID prevented increases in the expression of COX-2 and HIF-1*α* and in the production of IL-6 and TNF-*α*. Thus, these results suggest that ID effectively inhibits symptoms of colitis caused by DSS. 

Inflammatory cytokines such as IL-6, TNF-*α*, and interferon-*γ* mediate the pathogenesis of murine colitis [27–29]. Cytokines and chemokines are secreted by immune cells like T lymphocytes and macrophages that infiltrate the inflamed region. Studies in patients with UC have shown that gene [30] and protein expression [2–4] of IL-6 and TNF-*α* are similarly increased in the rectal mucosa. Consistent with the notion that IL-6 and TNF-*α* plays a causal role in the pathogenesis of UC, we found that ID suppressed the DSS-induced increase in IL-6 and TNF-*α* in mouse serum.

During the inflammatory process, the COX-1mRNA and protein activity do not change, whereas COX-2 levels increase dramatically, leading to the production of proinflammatory PGs [31]. However, Okayama et al. [32] found that both COX-1 and COX-2 inhibitors exacerbate inflammation and ulceration in the colon. Nevertheless, selective inhibitors like lumiracoxib have been developed as nonsteroidal anti-inflammatory drugs, many of which have been shown to be efficacious in a model of chemically induced colitis [33, 34]. 5-aminosalicylates, another drug class used to treat IBD, exert anti-inflammatory effects by inhibiting COX-2 activation [35]. Our study showed that ID inhibited the DSS-induced increase in COX-2 activation. These results suggest that the anti-inflammatory effect of ID is attributable to the regulation of COX-2 in DSS-induced colitis.

Inflamed mucosal tissue of colitic mice is highly hypoxic, leading to the overexpression of HIF-1*α* [36]. Clinical studies have also verified the upregulation of HIF-1*α* in colonic tissue from patients with IBD [37, 38]. And in HIF-1*α* overexpressing mice, Nuclear Factor-*κ*B activity and the expression of pro-inflammatory genes were sequentially elevated [39]. Moreover, HIF-1*α* was shown to directly bind to the COX-2 promoter, thereby regulating the expression of COX-2 protein in two colorectal carcinoma cell lines, HCT116 and HT29 [40, 41]. It is therefore tempting to speculate that in the present study, the DSS-mediated increase in HIF-1*α* expression triggers the upregulation of COX-2. By virtue of its ability to inhibit the increased HIF-1*α* expression, ID therefore suppresses COX-2 activation and the ensuing inflammatory response.

## 5. Conclusions

We have shown that ID reduces the clinical signs and levels of inflammatory mediators in DSS-induced colitis in mice. This study provides experimental evidence to show that ID might be a useful therapy in the treatment of UC.

## Figures and Tables

**Table 1:** Criteria for disease activity index.

Score	Weight loss (%)	Stool consistency	Bloodstain or gross bleeding
0	None	Normal	Negative
1	1–5	Loose stool	Negative
2	5–10	Loose stool	Positive
3	10–15	Diarrhea	Positive
4	>15	Diarrhea	Gross bleeding

**Table 2:** Criteria for assessment of microscopic rectal damage.

Score	Remarks
0	Normal colonic mucosa
1	Loss of one-third of the crypts
2	Loss of two-third of the crypts
3	Lamina propria covered with a single layer of epithelial cells with mild inflammatory cell infiltration
4	Erosions and marked inflammatory cell infiltration
